# Asking the experts: A focus group and review on eyewitness memory in the multicultural context of South Africa

**DOI:** 10.12688/openreseurope.19633.1

**Published:** 2025-02-24

**Authors:** Laura Anne Weiss, Eva A. J. van Rosmalen, Annelies Vredeveldt

**Affiliations:** 1 Social, Health and Organisational Psychology, Utrecht University, Utrecht, Utrecht, The Netherlands; 2 Optentia Research Unit, North-West University, Vanderbijlpark, South Africa; 3 Department of Criminal Law and Criminology, VU University Amsterdam, Amsterdam, The Netherlands

**Keywords:** culture, cross-cultural psychology; police interview; eyewitness memory; intercultural communication

## Abstract

**Background:**

The purpose of this study was to create a theoretical overview of cultural differences and intercultural communication in eyewitness interviews in a multicultural context. Specifically, we focused on the context of South Africa, one of the most culturally diverse countries in the world.

**Methods:**

We conducted a critical review of the relevant literature and organized a focus group with 12 international experts from different academic fields and cultural informants from South Africa to identify and deepen our understanding of cultural factors that may play a role in eyewitness interviews. We differentiated between eyewitness factors (e.g., emotional response) and the interaction between interviewer and eyewitness (e.g., miscommunication).

**Results:**

The main factors that emerged from the analysis were: individualism/collectivism, perception and presentation of facts, influence of demographics, presentation of self and personal style, subjective descriptions, emotional response, language, influencing behaviors in high-/low-context cultures, characteristics of the interaction and miscommunication. Each of these factors has a number of subcategories.

**Conclusions:**

The present article contributes to our understanding of cultural differences in eyewitness memory and intercultural communication by systematically collecting and analysing expert knowledge and situating it within the existing literature for the first time.

## Plain language summary

Interviewing an eyewitness to a crime is not a simple task. Often the eyewitness and police interviewer come from different cultural backgrounds. These cultural differences may influence the description given of a crime and the interaction between both parties in the interview; miscommunication and the withholding of information may be a result. This is especially relevant in South Africa, where many different cultures co-exist. To provide an overview of cultural factors relevant to this cross-cultural situation, we invited 12 experts from around the world to discuss two research questions specific to the South African context: (1) How do eyewitness reports differ between cultural groups? and (2) How do cultural differences affect the communication between the police interviewer and eyewitness? From the experts’ discussion, central themes emerged relevant to each research question.

For question 1, they found: (a) characteristics of the eyewitness report (like specificity and detail), (b) demographic perceptions (about gender and race) and (c) various eyewitness factors (their assumptions and motivations) to be relevant in answering the question. When discussing question 2, experts noted (a) various police factors (such as cultural sensitivity and police perceptions) and (b) the interviewer-eyewitness combination (such as demographic characteristics and interview dynamics) as factors that may affect communication between the parties in the interview. Our findings provide a framework for future research on eyewitness interviews in cross-cultural contexts. They also highlight important factors police interviewers, lawyers and judges should be aware of when an eyewitness is interviewed by someone from a different cultural background. As eyewitness testimony is often a crucial piece of evidence in criminal proceedings, it is imperative that everyone involved in the phases of a criminal prosecution understands cultural differences in eyewitness reporting of crimes and potential pitfalls in cross-cultural communication.

Memory is shaped by culture. Research has shown that culture contributes to how, when, and what people remember ( Wang & Ross, 2007). This is especially relevant in the field of international criminal justice. In this context, the term ‘culture’ can be understood as consisting of three categories: (1) the cultural equipment of language, tools, and skills, (2) the socio-cultural norms, and (3) culture-specific convictions about justice ( Almqvist, 2006). The present paper focuses on an area in which the influence of culture on memory can have far-reaching consequences: eyewitness memory.

Because the justice system relies heavily on eyewitness statements ( Wells *et al.*, 2006), criminal investigations and legal decision-making suffer if there are miscommunications between eyewitnesses and investigative interviewers or if judges or juries misunderstand or misinterpret eyewitness testimony. Especially in culturally diverse environments, it is important to take the critical role of culture in remembering and reporting events into account. South Africa is among the most culturally diverse countries, with four main population groups (black African, coloured, Indian/Asian, and white), comprised of a multitude of ethnic groups ( Statistics South Africa, 2020) and 11 official languages ( Mesthrie, 2002). Interestingly, South African police officers regularly conduct interviews in English, while most South Africans are not native English speakers ( Statistics South Africa, 2012). Research in such a multicultural context can help us understand how culture affects eyewitness memory and communication. This is particularly important considering that most research on eyewitness memory to date has focused on Western, Educated, Industrialized, Rich and Democratic (WEIRD) populations ( Henrich *et al.*, 2010). There is a clear need for attention to different cultural groups ( O’Brien & Kebbell, 2014; Powell & Bartholomew, 2003).

Previous research has revealed cultural differences in autobiographical memory (e.g., Wang, 2013a) and highlighted potential issues in intercultural communication in investigative interviews (e.g., Beune *et al.*, 2010), but the literature on the role of culture in eyewitness memory is fragmented and incomplete, particularly regarding African contexts. To investigate cultural differences in eyewitness memory in South Africa, we therefore combined a critical review of the relevant literature with the findings from a one-day focus group involving academic experts from different disciplines and cultural informants from different cultural groups in South Africa. Following the call of Wang and Ross (2007) for greater efforts to review the role of culture in memory from a multitude of theoretical perspectives, we invited experts from areas of psychology, sociology, criminology, law, and linguistics. We conducted a qualitative analysis of the rich data from the focus group and linked the identified factors to the findings from the critical literature review, to answer our research questions: *What should be taken into account when analysing (1) cultural differences in eyewitness memory and (2) intercultural communication between the interviewer and interviewee in South Africa?* To our knowledge, the present study constitutes the first attempt to provide a theoretical framework on eyewitness memory and intercultural communication in a multicultural context.

## Method

### Literature review

We conducted a *critical review* to investigate our research questions, which synthesizes material from diverse sources. Grant and Booth (2009) note that a critical review usually includes a degree of conceptual innovation and typically results in a model. This type of review takes stock of the previous body of work and provides a launchpad for a new phase of further research and testing of the suggested model.

We searched the literature for studies on cultural differences in memory reporting and intercultural communication in police interviews and critically evaluated them. We specifically looked for studies conducted with (South) African participants and studies on eyewitnesses but also included a broader array of studies, as the literature on our topic of interest is sparse. We synthesized insights from diverse areas of research. The results of the critical review, combined with the findings of the focus group, informed our final theoretical framework.

### Focus group

***Design and context.*** The purpose of the focus group was to identify cultural differences and possible communication problems in police interviews with eyewitnesses in cross-cultural contexts, with a special focus on South Africa. We used focus groups instead of one-on-one interviews because the group dynamics and social interaction may spark new ideas and create a synergy that generates richer data ( Rabiee, 2004). It allows a debate about complex phenomena such as race ( Cyr, 2016). Therefore, the focus group allowed us to gain new insights that were not available in the literature. The study received ethical approval from the Ethics Committee for Legal and Criminological Research (CERCO) on the 16 ^th^ October 2018 at VU University Amsterdam, the Netherlands. The study adhered to the Declaration of Helsinki. To preserve anonymity, experts are not named throughout the article, which is common practice for focus groups.

***Participants.*** The focus group consisted of 12 experts in fields related to our research questions: two cross-cultural psychologists, a developmental psychologist, a sociologist, a counselling psychologist, a researcher in law and political sciences, a researcher in defence and security, a work and organizational psychologist, a research psychologist, a social linguist, an international criminal law scholar, and a criminologist. Six participants were female, six male. They were from the Netherlands ( *n*=4), South Africa ( *n*=3), Cameroon ( *n*=1), Turkey ( *n*=1), China ( *n*=1), Bulgaria ( *n*=1), and the Czech Republic ( *n*=1). The three South Africans were from three different cultural groups (English, Afrikaans, and Xhosa) and served as cultural informants. Five other participants had worked or were currently working in South Africa.

We selected the experts based on their expertise. They were identified through our professional networks, by examining the literature, and by asking identified experts for referrals. To be included, they had to have published extensively on the topic of culture or memory ( *n*=5); published/focused their PhD study on an area relevant to our research questions (e.g., sexual violence in Africa) ( *n*=4); or function as cultural informants from South Africa with additional academic knowledge on African communities or related topics (e.g., crime, violence and victimhood in South Africa) ( *n*=3). Two additional South African experts were invited but were unable to attend. Instead, they provided written feedback on the manuscript. Five members of the research team were present during the focus group, all working in the field of eyewitness memory in cross-cultural contexts. The first author Laura Weiss, PhD (postdoc) acted as the moderator during the focus group. She had previously led focus groups. She knew five of the particpiants personally, and had e-mailed with all other participants previous to the focus group.

The participants knew that the researcher had previously worked in South Africa and that she was now conducting her postdoc on the topic of the role of culture in eyewitness testimony.

***Materials and procedure.*** The focus group took place at a Dutch university in February 2020. It was conducted in English and audio-recorded. First, the reseachers introduced themselves and the topic of the study. After participants had provided written informed consent, and introduced themselves, we presented the topic and illustrated it with anonymized text excerpts from real eyewitness interviews conducted by the South African police. Subsequently, we had a 2-hour whole-group discussion on the question: What to take into account when analysing cultural differences in eyewitness memory and intercultural communication between the interviewer and interviewee, specifically in South Africa? We used semi-structured questions, supplemented by subsequent probing questions. The discussion ended with a round of asking each participant about their insights. Next, participants split up into three small groups to create a coding scheme for analysing South African eyewitness interviews. For this exercise, they received four or five excerpts (different for each group) of interviews with male and female eyewitnesses from different cultures reporting on various crimes. After 1.5 hours, the group reconvened and presented their coding schemes to each other for the last half hour of the meeting. Field notes were made during and shortly after the focus group.

***Analysis of data.*** Data saturation was discussed and it was decided that we could continue with the analysis. We analysed the data of the focus group using qualitative thematic content analysis, following the four stages of qualitative data analysis: data preparation, familiarization, coding, and generating meaning ( Ruona, 2005). EVR transcribed the audio recordings of the plenary discussions verbatim and pseudonymized them. Next, the transcripts were inserted into Atlas.ti (data preparation). The first coder (LAW) started the coding process by reading the transcripts of the discussions and taking initial notes (familiarization). Both coders (LAW and EVR) coded the first 10% of the transcripts by assigning initial codes to every theme or topic. Codes consisted of a main code and, if needed, additional sub-codes and sub-sub-codes. The two coders resolved differences by discussion. The coders repeated this process twice until a third of the data was co-coded and discussed. The remaining transcripts were coded by LAW, who examined the list of codes to determine whether certain codes could be merged into meaningful overarching higher-order codes (coding). Finally, we used the output from the small-group exercise as a basis to structure the main codes (generating meaning). Although we coded everything, we only describe the concepts directly related to our research questions. We used member checking for verification, feedback, and validation of the content and interpretation of the findings from the participants. For this, we sent a draft version of the manuscript (but not the transcripts) to all participants to check, comment, and add to the results section if needed. This quality control process in qualitative research increases the (external) validity, credibility, and accuracy of the results ( Harper & Cole, 2012). We received feedback from ten experts. Two of the cultural informants checked specifically if the content concerning the South African background was described correctly. All feedback was integrated into the manuscript.

## Results

We combined the qualitative analysis of the results of the critical literature review with the findings of the focus group, because this integration will provide the most insight into how the findings of the two methods overlap. We have distinguished what came out of which method by use of references and experts’ quotes. An overview of the main and sub-factors for each research question is provided in Table 1.

**Table 1.** Overview of Factors Concerning Cultural Differences and Intercultural Communication in Eyewitness Interviews in South Africa.

Main factor	Subfactors
Cultural differences in eyewitness memory	
Individualism/collectivism	• Interdependent vs. independent self-construal • Concrete vs. abstract self-description
Perception and presentation of facts	• Perception and processing • Specificity & level of detail • Coherence • Focus • Sequence • Vicarious memory • Deception • Urgency to report
Influence of demographics	• Gender • Race • Socio-economic status • Age (norms)
Presentation of self and personal style	• Positioning • Eyewitness style
Subjective descriptions	• Storytelling • Subjectivity • Assumptions • Intensity of experience
Emotional response	• Expressing emotions • Trauma
Intercultural communication between interviewer and interviewee	
Language	• No shared native language • Language barriers • Language norms
Influencing behaviours in high-/low- context cultures	• Context-oriented & indirect vs. content-oriented & direct communication • Persuasion
Characteristics of the interaction	• Emotional and role congruence • Prejudices • Identification • Cultural knowledge • In-group out-group dynamics • Power dynamics • Saving face • Acquiescence • Perception of police
Miscommunication	• Importance of details of time and place • Concept of family • Taboo on discussing violence • Mystical beliefs • Nonverbal communication

### Terminology

The focus group first reflected on terminology: what words are acceptable or loaded in which context. For example, a European expert proposed ‘ *Call it ethnic identity. I am very happy with that term*’, and a cultural informant explained: ‘ *Not necessarily, because I think ethnic has not the same meaning in South Africa. The word ethnicity is loaded in South Africa, and I don’t think you can… I was very surprised at the loose use of ethnic here*.’ In South Africa, the word ‘race’ is commonly used to understand different groups, while the word ethnic ‘ *is used as a very problematic descriptor’* that usually denotes Africans. Because our research focuses on the South African context, the term ‘race’ was used in the remainder of the discussion and the present manuscript. This word may be regarded as inappropriate in other countries but was chosen to reflect the content of the discussion, as well as the South African context.

The focus group also discussed the term ‘ ***culture**’*, connecting it to race, language, colour, identity or socio-economic status (SES), or a combination of those. Non-material culture was linked to ‘ *things like norms, roles, beliefs, values*'. Others defined culture as cultural understandings of ‘ *how they are connected to certain cultural habits or certain origins of the people, […] functional definitions what it means to have this […] culture*.’ The critique of the term culture was that it is a broad and vague term. Hofstede’s (2011) theory was seen as a starting point. However, some regarded the individualism/collectivism dimension too broad to be useful and other dimensions as insufficiently supported by evidence. Nonetheless, all agreed that culture is a relevant and important concept in South Africa.

 Finally, the experts noted that eyewitness interviews involve not only bystanders but also victims. Throughout this article, we will use the term ‘eyewitness’ to include both bystanders and victims.

### Cultural differences in eyewitness memory

***Individualism/collectivism.*** Within cross-cultural psychology, an influential model constitutes Hofstede’s six dimensions of national cultures ( Hofstede *et al.*, 2010): power distance, uncertainty avoidance, individualism/collectivism, masculinity/femininity, long-term/short-term orientation, and indulgence/restraint ( Hofstede, 2011). The most well-known dimension is individualism/collectivism. Broadly speaking, Western societies are more individualist and African societies more collectivist, but there can be large variations within societies. Accordingly, some cultural groups in South Africa are more individualist (i.e., Afrikaans and British) and some more collectivist (i.e., black, Asian, coloured) ( Vogt & Laher, 2009).

The individualism/collectivism dimension is likely to play a role in eyewitness reports. First, memory reports are shaped by whether someone has an ***interdependent*** or ***independent self-construal***. People from collectivist societies tend to have a more interdependent self-construal, which means their identity is linked to the social roles they adopt in their families and societies ( Markus & Kitayama, 1991). As a result, they are more likely to retrieve aspects of memories related to their social identity and relationships ( Jobson & O'Kearney, 2008) and report more about the context and background of a scene ( Masuda & Nisbett, 2001). In contrast, people from individualist societies tend to have a more independent self-construal, which means their identity is linked to their own achievements and goals. Consequently, they are more likely to retrieve aspects of memories related to their personal identity and report specific elements of a scene ( Markus & Kitayama, 1991).

Individualism/collectivism does not only influence *what* people report but also *how* they report it, for instance, how specific and concrete their descriptions are ( Wang, 2001; Wang & Ross, 2005). Minimal research has been carried out on these topics in African contexts. In one of the few studies conducted in South Africa, Eaton and Louw (2000) found that African-language speakers from more collectivist societies produced more interdependent and concrete descriptions of themselves than English speakers from more individualist societies, whose self-descriptions were more independent and abstract. So, the individualism/collectivism dimension also leads to a difference between ***abstract*** and ***concrete self-description*** ( Eaton & Louw, 2000).

***Perception and presentation of facts.*** Both in the literature and the focus group, it was established that how eyewitnesses perceive and present information differs between cultural groups. First, ***perception and processing*** are influenced by culture. For example, Western Europeans scored higher on depth perception and visuospatial processing tests than their sub-Saharan African counterparts (e.g., de Bruïne *et al.*, 2018; Hudson, 1960; Rosselli & Ardila, 2003), which could affect eyewitness reports, for instance, when describing or identifying a weapon used during the crime.

During the focus group, it was remarked that the ***specificity*** of the report also differs across cultures: ‘ *People can report specific elements from the event as opposed to generic information*.’ This is closely related to the ***level of detail***, which was also considered an important factor: ‘ *We talked about how to code the detailedness of the narrative. So that has been established as something often influenced by cultural background*’. An example of detailedness that arose in the discussion was orientation information (i.e., does the eyewitness provide information about time and location?). The group discussed that the level of confidence and certainty about specific details expressed during the interview is likely linked to culture. The literature supports evidence for cultural differences in the level of detail provided in eyewitness statements. A recent study showed that Ghanaian mock eyewitnesses tended to report fewer details compared to Dutch eyewitnesses ( Anakwah *et al.*, 2020).

Furthermore, it was mentioned that the ***coherence*** of the narrative is reflected in causal connections in storytelling, for instance, by linking words such as ‘because’. Coherence can also be increased by adding orientation information, like temporal markers such as ‘yesterday’ or ’and then’. Finally, experts stated that coherence is reflected in how consistent the eyewitness’s story is and that these coherence indicators likely differ between cultural groups. Literature confirms that narrative coherence depends on cultural expectations ( McAdams, 2006). Whether a story is seen as coherent depends on the narrative traditions of a subculture, with each culture having different narrative traditions with its own standards of coherence ( McAdams, 2006).

Additionally, the experts discussed ***focus***. What or whom an eyewitness focuses on in their testimony could vary between cultures. The focus can be on the self, the perpetrator, the crime, the setting, or the object. However, it may also be affected by gender: ‘ *Men tend to only focus on the weapon. […] Whereas women would focus more on the person, not on the object*.’ Gender and culture may interact, with greater gender differences in certain cultures than others. Language may also influence the focus. A linguistic expert explained that *‘linguists categorize […] verb-framed languages versus noun-framed languages. […] Verb-framed means that children first learn action words, verbs. In some other languages, it is just the nouns. It introduces the focus. What to choose.*’

The group also discussed the importance of the ***sequence*** in which events are presented. The order in which eyewitnesses present their story suggests which aspects are most salient to them. In the exercise, the experts noticed that people present information in different ways, which may be linked to their cultural background. Communities in South Africa have their own specific oral storytelling traditions with their own constructs of story form, influenced by the cultural-linguistic context ( Reitmaier *et al.*, 2011).

Some eyewitnesses may report ***vicarious memories*** ( Pillemer *et al.*, 2015), which refers to reporting *‘things that you heard from someone else’* as if they happened to you personally. Literature confirms that in certain parts of Africa, eyewitnesses may talk about an event as if they were present themselves, while in fact, they are relaying what someone else told them ( O’Brien & Kebbell, 2014). The difference between seeing something yourself and hearing someone’s narrative is seen as insignificant. This cultural factor can introduce problems for fact-finding, for example, in international tribunals ( Combs, 2010).

Furthermore, the experts discussed ***deception*** by eyewitnesses. Some eyewitnesses may not speak the (full) truth, for example, to commit insurance fraud. The experts also noted that ‘messy’ or incoherent and factually inconsistent narratives could also indicate deception. Perceptions of and cues to deception have been found to differ between cultures ( Taylor *et al.*, 2015).

Experts also mentioned the ***urgency to report*** a crime:

*‘In middle-income communities, very minor crimes are reported and taken very seriously; whereas in low-income settings, characterized by ongoing violence, something has to be really obviously distressing before it is reported. So, harassment is likely not reported here; in fact, even rape has to be violently penetrative to be taken seriously.’*

A Xhosa cultural informant shared that he also did not feel the urgency to report after getting robbed, as he thought the police would not do anything anyway. The location of a crime also plays a role in the experienced urgency to report:

*‘Let’s say you are living in a very westernized, urbanized, middle-class context, something can be […] just a little bit violent to be reported. Whereas if you are working in Mitchell’s Plain, where such violent crime takes place, something has to be quite violent before it gets reported. So, I guess, eyewitness memory, it would be so traumatic, for a mugging maybe, whereas in Mitchell’s plain: ‘Well, that happens to me every day, I am not that traumatized by a mugging, I’ll get traumatized when I am almost attempted murdered. So, I think those, the urgency to report may also be based on those cultural differences.’*

The experts noted that the sense of urgency may increase when the crime is unexpected in terms of timing (i.e., in the middle of the day) or location (i.e., where crime takes place infrequently). This effect may be stronger in cultural groups that generally underreport. Lastly, the reason for filing a report may impact the sense of urgency. One of the cultural informants said: ‘ *I wouldn’t report it unless it was for insurance’*.

***Influence of demographics.*** Demographic characteristics but also broader demographic perceptions of gender, race, age, and socioeconomic status (SES) were discussed at length during the focus group. ‘ *Racial group, education, socio-economic status, all those are sociologic parameters. [… ] These are going to be background information for us to be able to understand all those interactions. […] All those are very complicated issues, multilayered issues.*’ ***Gender*** was mentioned throughout the discussion and connected to numerous topics. Gender roles differ between cultural groups, which in turn can affect how eyewitnesses report. They imply certain expectations, which are reflected in eyewitness testimony:

‘ *Participant (P) 1: A man will often in a narrative have a reason why they didn’t fight back; they have to justify. ‘There was a big gun.’ […] To kind of say, I am a man, my gender role expects me to be tough, but this time…*
*P2: I was drunk, I was inebriated.’*

In most cultures, it is more acceptable for women to identify as a victim than for men, but the gap between genders is greater in some cultures than others. Moreover, the experts stated that gender may affect memory reporting: ‘ *There are very consistent differences about how women recall events differently than men by, for example, including more details, more subjective information in the narrative*.’ Females also *‘use more adjectives to elaborate on things’.* In addition, ‘ *we know females have a much more diligent way of reporting*’. Indeed, empirical research has identified gender differences in memory and eyewitness testimony ( Areh, 2011; Herlitz & Rehnman, 2008; Horgan *et al.*, 2004).

Gender also affects how police interact with eyewitnesses. One expert noticed a gender difference in the transcripts in ‘ *the way that the females are being interrogated versus the males’:*

*The females, they just tell their story. And it is relatively consistent because they are not interrogated. And the males they are interrogated, and I don’t know because of this or because of other reasons, there is a lack of consistency in their stories*.’

Gender thus influences how eyewitnesses are interrogated, which in turn impacts how they respond and tell their story. One explanation for these differences is socialization: ‘ *South African men (black in particular) are socialized to talk less and do more, and as a result, their vocabulary is minimal when they are under pressure, such as when they have to report and recall the crime*.’ Hence, there is more probing during their interview. An expert on crime and gender issues added: ‘ *Black masculinity in South Africa is also very embedded in constructions of aggression, violence, power, sexual entitlement, etc.’*

Furthermore, ***race*** was discussed, including differing perceptions of one’s own and other people’s race and the racial stereotypes people hold. For example, a cultural informant described a prevalent stereotype in South Africa as ‘ *this notion that Nigerians sometimes are the criminals’*. Descriptions of the perpetrator’s race might differ between groups in South Africa because people do not want to portray themselves as racist. The fear of appearing racist may hinder direct and precise descriptions, for instance, when it results in using polite terms like ‘ *the coloured gentleman*’ when talking about a perpetrator from a different race. The perception of race in South Africa is influenced by the history of *apartheid*. As one cultural informant, who lives in South Africa but is originally from Cameroon, puts it: ‘ *We always look at the coloured or black people as the violent ones, the criminals. Whereas the apartheid history did play some kind of role in how things turned out*.’ When an interviewer and eyewitness are from different racial groups, the stereotypes and expectations they hold can hinder communication. Conversely, when the police interviewer culturally identifies with the eyewitness, this can facilitate communication.

One subgroup that worked on the exercise suggested constructing a matrix based on gender and race, for example, a white female interviewed by a coloured male. Such a matrix would allow for the discovery of certain patterns in communication across racial and gender groups ‘ *and the way these intersect in different ways’*. This is reminiscent of the concept of intersectionality, which emphasizes that different aspects of a person’s identity, such as race, gender, class, and religion, interact to create different modes of discrimination and privilege ( Cho *et al.*, 2013). For example, the level of discrimination suffered by a black woman is not simply the sum of discrimination against black people and discrimination against women.

Class, or more broadly ***socio-economic status***, is a factor that is hugely important in South Africa. Despite apartheid officially ending in 1994, the effects of apartheid education with racially segregated schools and under-resourced schools for black pupils is still evident in high levels of education inequality between racial groups and, as an effect, inequality in income ( Van der Berg, 2007):

P1: ‘ *Education in a country like South Africa […] has a lot of influence on communication styles. Also, what you dare to say to the police. If you are a university graduate, you will have a different attitude compared to those who come from a village with hardly any education*.’
  P2: ‘ *Something like cultural capital. Access to particular resources, knowledge systems*.’

Research has shown that eyewitness ***age*** consistently influences identification performance. For example, the elderly and young children perform worse than younger adults ( Wells & Olson, 2003). Certain cultural reporting patterns may interact with age ( Wang *et al.*, 2011). Yet not only age but also norms around age could play a role. Respect is associated with age and, as a result, it affects the interviewer-eyewitness dynamics:

‘ *For instance, when a crime is conducted at night and the victim is a young black or coloured woman, the police can ask her where was she going at night, in a sort of commanding role, or as if they are reprimanding her. Whereas if the victim who is reporting the crime is of almost the same age or older than the police, they would not ask such a question.’*

***Presentation of self and personal style.*** The experts considered ***positioning***, the way eyewitnesses portray themselves, to be meaningful:

‘ *When people tell a story, they have choices. When they speak about themselves, do they position themselves as victim, as actor, as survivor? To what extent is blame attributed? How do they position themselves relative to their attacker? […] All of those things, when you look at them you may find patterns.’*

As discussed during the focus group, the ***eyewitness style*** comprises nonverbal behaviours, linguistics and semantics. Some eyewitnesses use more assertive language, whereas others use more submissive language. This concerns how eyewitnesses typically phrase things, the flow of their narrative style, the length of their responses, and how direct they are. Autonomy is also an important factor during communication: does the eyewitness express feeling helpless or in control during the crime and interview?

***Subjective descriptions.*** ***Storytelling***, as seen in the contextualization, form, and structure of an eyewitness’s story, differs between cultures. Expectations towards the listeners in terms of feedback, such as expecting frequent interjections, can also vary. An interviewer who comes from a cultural background that favours fact-driven storytelling could feel overwhelmed by contextualized storytelling and may perceive the story as unfocused and rambling ( Cook-Gumperz & Gumperz, 2002), and possibly perceiving the eyewitness as untrustworthy.

The group discussed that the ***subjectivity*** of memory reports may culturally differ. One expert described her research on this topic:

‘ *We actually did a study comparing parents’ (mothers’) and children’s recall of the same events. They experienced the same events, but we interviewed them separately. They shared more similarity in terms of facts, but they diverged more in terms of subjective matters. […] There is also interesting cross-cultural differences. […] Mostly. you can see how different perspectives can diverge or converge and also that can be influenced by culture*.’

According to the experts, people from different cultural groups can also have different ***assumptions*** about the world and others. Sometimes, people make statements based on those assumptions, which includes guessing. For instance, in one excerpt, the perpetrator had a scar and the eyewitness said: ‘He probably picked that up in a fight’.

***Intensity of experience*** was discussed as well. Eyewitnesses may differ in the expressed intensity with which they experienced the crime (e.g., ‘It changed my life forever.’ vs. ‘Oh, it didn’t matter that much for me. I recovered quickly.’), which may vary depending on gender roles and cultural background.

***Emotional response.*** Emotions during and after the crime were discussed extensively. The extent to which people ***express emotions***, feelings, and desires differ between cultural groups, which, in turn, affects memory. People from cultures where independent self-descriptions are more common (usually individualist cultures) described their memories in a more emotionally elaborate and expressive way than people from cultures where interdependent self-descriptions are more common (usually collectivist cultures) ( Wang, 2001). The experts echoed this finding: ‘ *There is kind of semantic memory, and the emotional aspect is huge.*’ In addition, ‘ *the elements can be emphasized from memory, depending on trauma’*. How trauma is expressed ‘ *varies greatly in terms of people who do not have the context and ability to discuss it in terms of white middle class, Western psychology*.’ People who do not possess that terminology may rather speak in terms of physiological symptoms instead of emotions or psychological symptoms. A cultural informant said: *‘I know some African languages do not even have the word for depression*.’ They would rather speak about ‘ *their stomachs, lower back*’.

***Trauma*** was also mentioned as impacting eyewitness testimony: ‘ *If they are traumatized, they could have already developed some PTSD symptoms. Of course, that can influence the specificity of the recall.*’ Trauma may be rooted in earlier experiences and exposure to crime, which may differ between cultural groups. A crime may be ‘ *more traumatic if you have not been exposed before*’. How trauma is understood and whether it is taken seriously depends on socioeconomic status:

*Cultural informant (C) 1: I think also you need to understand trauma in South African context. That it’s really taken seriously in the middle-upper classes that they are actually traumatized. But in the much poorer neighborhoods, it is not a thing. It is not taken seriously. Many police stations do not have any trauma support. So, it’s kind of attached to some social status and income level.*
*P2: So, the people are not traumatized in the poor neighborhoods or their problems not taken seriously?*
*C1: They are the most traumatized. If you get mugged and then two days later you see someone get mugged, you are re-traumatized again. So basically you are living in a constant state of fear. So there is a lot of trauma there, and it is not taken into consideration by themselves, as well as by the police. This is largely due to lack of mental health awareness and literacy in the poorer communities, even though they experience more mental health issues.*

### Intercultural communication between interviewer and interviewee

***Language.*** Groups have their own ‘ *culture of telling, culture of explanation’*. Therefore, the quality of the interaction may depend on language, especially when interlocutors have different native languages. The experts observed in the excerpts that ***no shared native language*** between the interviewer and eyewitness can lead to miscommunication and inaccuracies. The experts noticed in the excerpts that the interviewer patiently waits for an answer when communication is smooth. Yet when difficult to communicate with the eyewitness, the interviewer often becomes more directive and starts to fill in the blanks. Importantly, English is not the native language for most South Africans and is only spoken by 9.6% of South Africans as their first language ( Statistics South Africa, 2012). Yet many interviews are conducted in English, which could lead to ***language barriers*** for both interviewer and eyewitness. Those barriers may affect the coherence and detailedness of the statement. Even when a translator is present, ‘ *some things do get lost in translation sometimes […]. People use euphemisms when they can’t really speak or describe whatever went down.*’ Furthermore, ‘ *language of narration differs dependent on the interlocutor’*. Eyewitnesses talk differently to friends and family than to the police, where they are held accountable for what they report.

Memories and self-narratives are mediated by spoken language, which serves as a vehicle for culture ( Marian & Kaushanskaya, 2004). ***Language norms*** underlying the multiple languages of South Africa became apparent in a study by Thetela (2002). In Xhosa and Southern Sotho, there is a language of respect for women (called *hlonipha* in Southern Sotho). In conversations regarding sexuality, *hlonipha* ensures politeness through euphemisms, being vague, and avoiding profanities. The linguistic code used by rape victims to maintain their cultural gender identity may undermine their legal cases for several reasons. First, the code could be perceived as carrying an underlying meaning of consent. Further, police interviewers may require that eyewitnesses use explicit language (e.g., to describe genitalia), but eyewitnesses may be constrained by the *hlonipha* code from complying with such requests. The embarrassment of being asked to use sexually explicit language may severely influence eyewitness testimony. Incomplete statements, evasive answers, silence, crying, and hesitating can, in turn, lead to a negative attitude of interviewers towards the eyewitness, which can result in impatience, interruptions, rudeness, and victim-blaming by the police ( Thetela, 2002). This indirect and vague way of talking about sexuality may thus negatively affect statements of female rape victims.

***Influencing behaviours in high-/ low-context cultures.*** One important distinction in communication styles is between high- and low-context cultures ( Hall & Hall, 2000). In high-context cultures *, **communication*** is more ***context-oriented*** and ***indirect***. In low-context cultures, communication is more ***content-oriented*** and ***direct***, and a higher value is placed on logic and deductive thinking ( Gelfand & Dyer, 2000). Deliberate actions of an interviewer to alter the recipient’s attitudes or behaviour (i.e., influencing behaviours) in police interviews have a different effect depending on someone’s communication style ( Beune *et al.*, 2009; Beune *et al.*, 2010). Research with suspects from low-context (Dutch) and high-context (Moroccan) cultures showed that rational ***persuasion*** was more effective in getting case-related information from the Dutch interviewees than from the Moroccan ones in police interviews ( Beune *et al.*, 2010). Being kind works particularly well for the high-context suspects ( Beune *et al.*, 2009).

***Characteristics of the interaction.*** Characteristics of the interaction between the interviewer and eyewitness must be taken into account when analysing communication:

‘ *There might be an **emotional congruence** or not (where a police officer replies adequately to a displayed emotion by an eyewitness), there might be **congruence** or not **in the roles** the two interactors take (in terms of hierarchy, for example).*

The level or lack of ***cultural knowledge*** and sensitivity toward the other person’s culture affects how the interview progresses. The level of emotional literacy or empathy is crucial for understanding the other person and their background. To what extent do they identify with the other and their culture, and can they relate to the other’s experiences? Culture can impede identification and understanding. However, when both come from the same cultural group, they can share cultural meaning:

*‘A shared meaning about a cultural space or the types of people in those cultural spaces, whereas if those two people are from completely different cultural backgrounds, the understanding is: “You don’t understand my victimization” […]. And I think those could be quite different narratives.’*

Another expert added that ‘ ***in-group out-group dynamics’*** should be considered: ‘ *If you define someone as out-group, first of all, perception homogenizes. So, it’s that stereotype of ‘they all look alike to me’. […] You don’t perceive your in-group as that homogenous.’* Out-group thinking also affects ***power dynamics***. One expert remarked: ‘ *You may think about looking at the power dynamic between the victim and the police, how that influences the richness of the information provided.*’ Other significant indicators of power dynamics are victim blaming and victim worthiness (e.g., a white woman is seen as more deserving of attention than a black man). Regarding victim-blaming, cross-national differences were found in the attribution of blame toward partner violence among respondents in South Africa, Nigeria, and the US ( Fakunmoju *et al.*, 2015). Respondents from the US were less likely to attribute blame for partner violence to the female victim than South African respondents.

A negative aspect of power dynamics, mentioned during the focus group, was further victimization by the police:

‘ *A black woman, she is more likely to be further victimized by the police. Like “Your dress was too short.” or “You were walking late at night, what were you expecting?” Or if it is domestic violence: ”Go home and solve your things with your husband”.’*

The experts discussed the role of authority as well. Signs of police authority can be observed in the use of corrective or condescending language:

*‘Whether the police officer is reinforcing what the interviewee is saying or negating it or just being apathetic and dismissing it. Arrogant tone or a condescending tone, which is obviously linked to “Do I believe you?” and “Are you a plausible victim”?’.*

Eyewitnesses may react to the power dynamics with submissive language. The power dynamics can also affect how comfortable eyewitnesses are reporting the details of their experiences, especially traumatic ones. Sometimes, expectations of further victimization by the police may lead to not reporting a crime at all. In South Africa, violence against women is common but underreported due to a lack of trust in the criminal justice system ( Gordon & Collins, 2013).

Issues of face, or the motivation of an eyewitness to be perceived positively, may apply in specific instances, such as interviews with rape victims. Eyewitnesses may leave out or change information if they believe this will portray them in a better light. In some cultures, the need for ***saving face*** when disclosing certain personal information is more prominent than in others ( Taylor *et al.*, 2015). The experts mentioned that different meanings exist within cultures about what face loss means, which may impact if and how eyewitnesses talk about events. When eyewitnesses are concerned that certain aspects of their experience may be seen as shameful, they will talk about these aspects differently (or not at all), hindering communication.

Another cultural difference affecting communication discussed by the experts is social desirability and ***acquiescence***. Eyewitnesses may want to please the police:

*‘P1: Then there is social desirability. […]*
*P2: Something like: “What does the police expect me to say?”*
*P3: I think we noticed in the data that sometimes an eyewitness would be saying something, and the police would then change it, and then they would just say: “Yes, yes, that.”*
*P1: Probably that also reflects the power dynamic in a way by trying to please the authority in the context.’*

People from different cultures differ in their expression of social desirability ( Valchev *et al.*, 2014) and their tendency to agree with questions (regardless of content), known as acquiescence response style ( Van Herk *et al.*, 2004). This is likely due to cultural differences in agreeableness ( Johnson *et al.*, 2005) and obedience to authority ( Taylor *et al.*, 2015). Specifically, in South Africa, black participants scored higher on positive impression management than white participants ( Valchev *et al.*, 2014). The desire to agree with the interlocutor, especially an authoritative one, may outweigh the desire to provide an accurate answer ( Markus & Kitayama, 1991). In line with these findings, de Bruïne *et al.* (2018) found that sub-Saharan African participants were significantly more likely to respond ‘yes’ during an object recognition test than their matched Western European counterparts.

The ***perception of police*** was an important topic of discussion throughout the focus group. Mistrust in the police could lead to various communication problems. When there is no trust in the system, the eyewitness may not confide in the police, and important information may be withheld. Being afraid of a breach of confidentiality may dissuade an eyewitness from mentioning the name of the perpetrator. The main perception of the police is that they cannot be trusted and ‘ *that they are never going to do anything anyway*’. This perception was mentioned eight times by different experts. Although a cultural informant described that South Africans from all groups perceive the police as incompetent, distrust in the police may be even more pronounced in some ‘ *black and coloured culture’:*

‘ *Because generally, the stats in South Africa show that those two cultures are the most likely to be victims of crime. Because they have seen so much crime and nothing has happened, their trust in the police is very low*.’

***Miscommunication.*** When a police interviewer communicates with an eyewitness from a different cultural background, as is common in South Africa, miscommunications are likely ( Chick, 2002). Miscommunication due to cross-cultural differences can occur during interactions such as small talk, emphasizing, persuasion, ultimatums, and resistance ( Taylor *et al.*, 2015).

Combs (2010) describes several examples of differing cultural understandings that impede communication in international criminal fact-finding. In some cultures, ***details of time and place*** are seen as unimportant. Therefore, eyewitnesses may react impatiently when asked about such details. Another difficulty is figuring out if and how people are related to others, as the ***concept of family*** is much broader in some non-Western countries. For example, the African way of describing a friend as ‘sister’ or ‘brother’ can lead to confusion. Furthermore, in some conflicted African regions, there is a ***taboo on discussing violence*** that happened in the past. People fear that doing so will cause violence to return, or view violence as suspicious, since talking about it is believed to aggravate social tensions.

These beliefs can cause eyewitnesses to evade certain questions, especially about sexual violence. For example, reporting rape in Rwanda and Sierra Leone can lead to stigmatization, ridicule, and rejection of the victims. In certain (South) African cultural groups, Christian belief is combined with African Indigenous beliefs, including beliefs in spiritual forces, ancestors, and witches ( Makgahlela, 2020). Superstition and ***mystical beliefs*** can lead to problems in legal fact-finding. For example, allegations of witchcraft, behaviour based on the perception of a bad omen, or testimony of magical powers can cause misunderstandings, especially for Western people who are unfamiliar with these concepts ( Combs, 2010).

South Africa has its own specific communication issues. One source of misunderstanding in this context is ***nonverbal communication*** ( Ntuli, 2012). In traditional African communities, some nonverbal behaviours are considered disrespectful, especially towards an older person. Examples include pointing at someone with one finger, looking someone straight in the eye during a conversation, remaining standing in the presence of senior people, and using the left hand when receiving something. White South Africans may find the opposite disrespectful, such as sitting down without being offered to do so. These differences have significant implications for the interpretation of eyewitness testimony, for instance, when an African eyewitness’s lack of eye contact, meant as a sign of respect, is interpreted as a cue to deception by a white interviewer.

Combs (2010) concludes that, although there are multiple barriers to understanding and assessing the credibility of eyewitness testimony from different cultures, there has been little effort to investigate systematically how cultural differences impact the understanding of such testimonies. In Table 1, we present an overview of the concepts that were discussed in the focus group and found in the literature review to provide the first systematic summary of cultural differences that impact testimony and communication between police interviewers and eyewitnesses in South Africa.

## Discussion

The purpose of this study was to create a theoretical overview of cultural differences and intercultural communication in eyewitness interviews in the multicultural context of South Africa. As the available academic literature is so incomplete and fragmented that it does not provide sufficient information, we organized a focus group with a diverse and multidisciplinary panel of international experts to identify and discuss relevant cultural factors. The theoretical insights from the experts and practical knowledge of cultural informants from different South African population groups provided a rich basis for our analysis of how culture influences how eyewitnesses report and interact with the police interviewer. Many of the findings of the focus group mirror findings reported in the literature.

First, the experts’ critique of the concept of ‘culture’ as too broad and vague is in line with the discussion in cross-cultural psychology about the meaning of culture. Kashima and Gelfand (2012) state that ‘culture is one of the most used and perhaps abused concepts in contemporary academic and popular discourse’ (p. 499). Accordingly, there are countless definitions of culture. Jahoda (2012) found that definitions of culture differ to a great extent; culture is defined as external, internal, or both, or through groups of several definitions. Many definitions are logically incompatible with each other. Therefore, he suggests explaining precisely what you mean by ‘culture’ in your particular situation and context, which we aimed to do.

 With respect to demographic perceptions, one thing that was mentioned many times by several experts in our focus group was the role of gender, which has also been emphasized in the literature ( Thetela, 2002; Wang *et al.*, 2011; Wang, 2013b). Gender and gender perception play a role in many of the other factors that relate to culture. Therefore, gender is an essential factor to take into account in any analysis of cultural differences and intercultural communication. This observation underscores that we cannot and should not look at cultural differences in isolation.

 Interestingly, Hofstede’s famous cultural dimensions ( Hofstede, 2011) were barely discussed during the focus group, and the experts considered individualism/collectivism too broad to be useful. Indeed, Hofstede reports only a single value for each country for each of the cultural dimensions. ^1^ Such a generalized view is of little value to the present study, which focuses on cultural differences within one country rather than average differences between countries.

With respect to intercultural communication in interviews, our findings are in line with reports that multiculturalism and diversity present significant challenges for the South African Police Services (SAPS) ( Buntman & Snyman, 2003). Specifically, the rural/urban divide, the differences in languages and cultural norms, tradition versus modern views, racial conflicts, immigration, and ideas on gender and sexuality are likely to result in misunderstandings. Thus, the communication process is not only influenced by culture in general but also by the specific police culture. In our focus group, a recurrent topic was the distrust that the population has in the police. Interestingly, not only the public perceives the police as ineffective. Both within the SAPS and the public, the widely held perception is that many of SAPS’ leaders and members are corrupt ( Newham & Faull, 2011).

Some of the challenges related to intercultural communication could be mitigated by training interviewers ( O’Brien & Kebbell, 2014). Powell and Bartholomew (2003) formulated guidelines for forensic professionals to communicate effectively with people from different cultural groups, such as establishing rapport with culturally appropriate greetings, avoiding potentially offensive terms and behaviour and being aware of cultural codes and typical cultural differences in intonation and nonverbal cues like eye gaze. Interviewers should acknowledge the broader socio-cultural context of both the crime and the response of the interviewee. Similarly, the focus group came to the conclusion that cultural sensitivity and emotional competence of police interviewers is crucial for effective communication and recommended using techniques that encourage free recall by using open questions. Most police officers in South Africa do not receive (sufficient) training and guidance to develop these skills ( Buntman & Snyman, 2003), highlighting the need for openly accessible guidelines for police interviews.

 One of the focus groups’ strengths was that the experts arrived at new insights by building on each other’s different fields of knowledge, in contrast with most of the literature approaching the topic from the perspective of a specific field ( Wang & Ross, 2007). The interdisciplinary theoretical insights were combined with the personal experiences of the cultural informants from different population groups (Afrikaans, Xhosa and English), as well as Africans and Europeans who have lived or currently live in South Africa, adding the perspective of foreigners in South Africa. The cultural informants were an important part of the discussion. They frequently talked about their own experiences, which gave narrative embodiment to theoretical concepts or ignited the discussion of a new theory or concept. While many of the concepts have been described in the literature, most of the literature does not refer to African countries, let alone South Africa. The focus group added many valuable insights about applying the theory to the specific multicultural South African context, while most cross-cultural studies compare countries.

 One limitation of the present study is that critical literature reviews use a less systematic approach than some other types of reviews and could therefore miss certain articles. However, our focus was not on conducting an exhaustive search but on critically reviewing and interpreting the conceptual contribution of the broad literature. In line with the aim of a critical review, the results are seen as a starting point for future research rather than as an endpoint ( Grant & Booth, 2009). Second, although we included professionals with a variety of different backgrounds, no police officers from South Africa were present. We invited two (former) police officers from South Africa, but they were not able to attend due to their workload. It would have been illustrative to hear about their experiences as interviewers. Nevertheless, we reached the main aim of the study to develop a theoretical overview based on the scientific knowledge of experts. Police interviewers will be more involved in future research, including the development of practical recommendations for the police. Third, we conducted only one focus group, while some researchers recommend at least two groups ( Carlsen & Glenton, 2011). Additional focus groups with other experts could have led to different results. However, we reduced the risk that we may not have found all relevant factors by combining the focus group results with the literature review. Last, while focus groups with experts can provide valuable ideas, it is important to acknowledge that the insights are not infallible. Nevertheless, the primary aim of the study was to obtain novel perspectives and knowledge, and the present theoretical framework forms a knowledge base that can be expanded upon and changed by accumulating additional insights.

## Conclusion

Detailed and accurate reports from eyewitnesses are vital for the successful functioning of the justice system ( Wells *et al.*, 2006). The present article contributes to our understanding of cultural differences in eyewitness reports and intercultural communication in South Africa by systematically collecting and analysing expert knowledge and situating it within the scientific literature. The findings provide a theoretical framework for future research on eyewitness interviews in cross-cultural contexts. In combination with findings from other studies, our findings also provide useful input for police interview guidelines, such as the Méndez Principles ( Méndez *et al.*, 2021), which will be addressed in future publications. For example, interviewers could assess beforehand whether the interviewee comes from a low- or high-context culture and decide which type of communication is likely to be more effective ( Vredeveldt *et al.*, 2023). Learning about cultural differences and potential pitfalls in communication can help police interviewers obtain better evidence in cross-cultural contexts and help legal decision-makers to evaluate that evidence in a culture-sensitive manner.

## Ethics and consent

The study received ethical approval from the Ethics Committee for Legal and Criminological Research (CERCO) on the 16 ^th^ October 2018 at VU University Amsterdam, the Netherlands. The study adhered to the Declaration of Helsinki. The participants received written information about the study, had time to ask questions, and then signed a written informed consent.

## Data Availability

The data (transcript of the focus group)_is restricted, as participants are anonymous. The data could make it possible to connect what is said to the identity of the participants. However, if certain parts are needed, it is possible to provide someone with parts of the data (contact a.vredeveldt@vu.nl), where the parts that could lead back to particpants’s identity are removed. The Ethics Committee for Legal and Criminological Research (CERCO) at VU University Amsterdam did not provide statements on data sharing.

## Footnotes

^1^ The values that are reported for South Africa are based exclusively on the white population, which only makes up circa 9% of the population
