# Supplementary material for: Asking the experts: A focus group and review on eyewitness memory in the multicultural context of South Africa
Source: Open Res Eur. 2025 Feb 24;5:54. [Version 1] doi: 10.12688/openreseurope.19633.1 (PMC11979577; doi:10.12688/openreseurope.19633.1)
Supplement: Peer review history

# Reviewer report: Reviewer response for version 1

**DOI:** 10.21956/openreseurope.21236.r51786

**Published:** 2025-04-08

**Authors:** Richard Kemp

**Affiliations:** 1 University of New South Wales, Sydney, Australia

This article describes the results from a one-day workshop / focus group designed to investigate cross cultural factors in eyewitness memory and eyewitness interviewing within a multi-cultural context – with particular attention to the South African context. The focus group was attended by 12 experts drawn from a number of academic disciplines, including psychology, law, sociology, political science, social linguistics and criminology. The experts were of several nationalities, including three from South Africa who were described as “ *from three different cultural groups (English, Afrikaans, and Xhosa) and served as cultural informants”*.

 The authors structured the discussions around two broad themes 1) factors relating to the eyewitness, and 2) factors related to the interaction between interviewer and eyewitness. Under these broad headings they drew out a number of factors and sub-factors which were identified by the experts as potentially impacting the information provided by a witness operating in a cross-cultural environment. The authors concluded that the outcomes of this focus group “ *contributes to our understanding of cultural differences in eyewitness memory and intercultural communication*” and suggest that “ *The findings provide a theoretical framework for future research… [and] useful input for police interview guidelines*”.  

 I was pleased to be invited to review this article as it focuses on a topic which has received very little attention. Eyewitness memory and eyewitness interviewing have been the focus of an enormous volume of research in the last 3 or 4 decades. Almost all of this research has been undertaken by researchers in a handful of developed counties, and even within these countries almost no attention has been given to cross-cultural factors which might play an important role in determining the quality of the information that can be obtained from an eyewitness. I was pleased to see these researchers turn their attention to this important and largely ignored issue.

 Given this I was a little disappointed that the research involved a focus-group of experts, only three of whom came from South Africa. Furthermore, as far as I can tell, none of these experts are likely to have had direct lived experience of being in the position of having to provide an eyewitness account of an incident in a cross-cultural context. I was also disappointed to see that the researchers were unable to arrange for any police officers to attend the focus group. I think an ideal arrangement might have been to hold a series of these focus groups, one “expert” group, one group attended by people with direct experience of being interviewed by police in this context, and one by police officers with experience of working in this context. By triangulating in this way, I think we might have identified some significant issues which may not be obvious to the “experts”. For example, I suspect that the experts may have not given sufficient attention to the issue of whether eyewitnesses in some contexts might choose not to report an event, or might choose not to make themselves available to police, due to a lack of trust with the authorities stemming from negative personal experience and/or cultural understanding and power imbalance.

 To be clear, I commend the authors for recognizing this significant gap in our understanding of issues that might impact the quality of eyewitness evidence. I also recognize that it can be hard to research these issues and I think that the focus-group approach has some merit as a starting point. However, I am a little disappointed by the rather limited scope of what has been achieved. I share the authors hope that this work will help to highlight the need for research in this area, and look forward to seeing this issue picked up by other researchers in the coming years. In particular I hope that in this future work we listen to those who have direct experience of cross-cultural interactions with police and that we find ways to incorporate this knowledge into police interview procedures so that we can improve the quality of eyewitness evidence and thus contribute to the operation of a fair criminal justice system.

If applicable, is the statistical analysis and its interpretation appropriate?

Not applicable

Is the study design appropriate and is the work technically sound?

Yes

Is the work clearly and accurately presented and does it engage with the current literature?

Yes

Are the conclusions drawn adequately supported by the results?

Partly

Are sufficient details of methods and analysis provided to allow replication by others?

Yes

Are all the source data and materials underlying the results available?

No source data required

Reviewer Expertise:

Eyewitness memory. Eyewitness identification. Identity verification. Forensic science. Expert evidence. Jury decision making.

I confirm that I have read this submission and believe that I have an appropriate level of expertise to confirm that it is of an acceptable scientific standard.

# Reviewer report: Reviewer response for version 1

**DOI:** 10.21956/openreseurope.21236.r51549

**Published:** 2025-04-08

**Authors:** Nkansah Anakwah

**Affiliations:** 1 Psychology, Birmingham City University (Ringgold ID: 1725), Birmingham, England, UK

Thank you for the opportunity to review the manuscript ‘Asking the experts: A focus group and review on eyewitness memory in the multicultural context of South Africa’. In this study, the authors conducted a focus group discussion with 12 experts and also undertook literature review on cultural differences and intercultural communication on eyewitness memory. Themes/ sub-themes that emerged for cultural differences in eyewitness memory reports included individualism-collectivism, influence of demographic factors, perception and presentation of facts, and subjective descriptions. For inter-cultural communication between interviewer and interviewee, themes that emerged included language, miscommunication, influencing behaviours in high-low context cultures, and characteristics of the interaction.

 The study is very interesting and addresses an important area, as there is a dearth of literature examining eyewitness memory in multicultural/ cross-cultural contexts. The paper is well written, original and makes an important contribution to the literature. Nevertheless, I’ve made the following comments/ suggestions for the authors to consider in improving the paper.

 The methods for the literature review is quite brief. The authors should consider providing more details regarding the methodology by highlighting how literature reviewed were gathered (i.e., how they identified and selected the literature) and any inclusion/ exclusion criteria.

 It was mentioned that excerpts from real-life interviews conducted by the South African police was used to illustrate the topic. How were the real-life witness interviews used to illustrate the topic/ what instructions were the experts given? Did the interview excerpts contain particular (cultural) issues pertinent to the topic for the focus group discussion? The authors can shed more light on this.

 Please clarify in the methods whether the focus group discussion took place in-person, online or whether it was a hybrid.

 One of the themes identified in the focus group discussion is the sequence of information, with cultures suggested to differ in the order information is presented. This theme can be discussed further, in relation with previous literature. It would also be good to provide example of how the order of presenting information may differ culturally.  

 The theme on vicarious memories can also be developed a bit, particularly within the context of relevant theory. Considering previous research shows collectivism / interdependent self-construal is linked to the acceptance/ reporting of information from social sources within African contexts (and other collectivistic cultures), the authors can expand discussion on this theme, highlighting the role of collectivism/ interdependent self-construal in vicarious memories.  

 It was highlighted deception cues have been found to differ across cultures. It would be good to discuss this further by highlighting what the literature shows regarding cultural differences.

 It was sometimes not explicitly stated whether some of the identified themes emerged from the focus group discussion or the literature review. This includes themes on influencing behaviours in high/ low context cultures and miscommunication.  

 It was mentioned in the discussion that the individualism-collectivism dimension is too broad to be useful in the study. This conclusion seems quite contradictory, in view of the fact that many themes/ sub-themes presented from the literature review and focus group discussion aligned with the individualism-collectivism dimension. For example, the independent-interdependent self-construal, cultural differences in specificity/ level of detail, vicarious memories, emotional response, acquiescence, and influencing behaviours in high/ low context cultures, are some of the themes from the current study that the literature shows align with the individualism-collectivism cultural dimension. Given the theoretical alignment of the individualism-collectivism dimension with some themes in the results, I find the dimension to be relevant to the work.  While the dimension has its limitations, it provides a framework for understanding the role of culture in behaviour and psychological processes. Considering the focus on the work was in a multicultural context, it would be expected that there would be variations in orientation towards individualism-collectivism.

If applicable, is the statistical analysis and its interpretation appropriate?

Not applicable

Is the study design appropriate and is the work technically sound?

Yes

Is the work clearly and accurately presented and does it engage with the current literature?

Yes

Are the conclusions drawn adequately supported by the results?

Partly

Are sufficient details of methods and analysis provided to allow replication by others?

Partly

Are all the source data and materials underlying the results available?

Partly

Reviewer Expertise:

Cultural influences on eyewitness memory, investigative interviewing in cross-cultural contexts

I confirm that I have read this submission and believe that I have an appropriate level of expertise to confirm that it is of an acceptable scientific standard, however I have significant reservations, as outlined above.

# Reviewer report: Reviewer response for version 1

**DOI:** 10.21956/openreseurope.21236.r51782

**Published:** 2025-04-07

**Authors:** Alyssa Jones

**Affiliations:** 1 Tarleton State University, Stephenville, USA

In a qualitative, but theoretically driven, focus group study, the researchers provide an overview of cultural differences and intercultural communication in eyewitness interviews in the multicultural context of South Africa. The researchers were interested in narrowing in on South African culture because it is one of the most culturally diverse countries, with four main population groups, comprised of a multitude of ethnic groups and languages. Additionally, South African police rarely use English when conducting an interview, although most South Africans are not native English speakers. The authors also point out that most cross-cultural studies compare countries to each other, but comparing within a country like South Africa may be necessary for applied issues such as those found within the field of Psychology and Law. I commend the researchers for tackling a complex aspect of such an important applied issue (eyewitness evidence gathering procedures) with the potential of having serious implications. Language and communication are difficult to study experimentally for numerous reasons, especially when memory and crimes (i.e., a situation associated with dynamic emotions and motivations involved) are involved. It is refreshing to read a new approach to investigating eyewitness evidence.

 The focus group consisted of international experts from a diverse range of multidisciplinary fields, who were asked to identify and discuss relevant cultural factors that may influence differences in communication across eyewitnesses with varying backgrounds and how these differences may influence the way these eyewitnesses report and interact with a police officer during an interview. To sum up their results, the authors conclude the following: (1) the concept of culture appears to be too broad, with many definitions depending on cultural context, (2) gender and gender perception play a major role in driving cultural differences in communication, (3) rural/urban divide, differences in languages and cultural norms, tradition versus modern views, racial conflicts, immigration, and ideas on gender and sexuality are likely to result in misunderstandings, including the specific police cultural involved in the communication process, (4) South African Police Services (SAPS) is perceived as highly ineffective and untrustworthy.

 A strength of the current study, as mentioned by the authors, is that their approach allowed the experts in the focus group to build on each other’s knowledge and co-create new theoretical insights combined with information from personal experiences. However, a limitation of the study is that the authors chose to conduct a critical literature review, which means they used a less systematic approach compared to other reviews and may have missed certain articles that exist in the literature. But the authors are transparent about their goal of creating a starting place for future research rather than an endpoint in the literature. Another limitation is that no real South African police officers were included in the focus study, which is a major limitation in my opinion because the rationale for the study includes South African police culture and communication. Finally, this study only includes one focus group instead of multiple focus groups like suggested in the literature (Carlsen & Glenton, 2011). But I cannot really speak to this limitation because I do not conduct focus group research and felt the authors did a great job at trying to ensure this did not negatively affect their findings (i.e., connecting the focus group's discussion to the existing literature rather than having additional focus groups within this study). 

 Overall, the work is clearly and accurately presented. The authors are clear and succinct in their review of communication differences found across cultures and how these differences may influence memory. In doing so, it really hammers home why this study is an important contribution to the applied issues in eyewitness memory and identification literature, as it is important to consider not only how the interviewer and interviewee communicate while also considering how this may influence the eyewitness in successfully identifying a guilty suspect from a police lineup. I feel the work clearly engages with the current literature, as the authors point out relevant research from the past 10-20 years throughout the paper and mostly connects these findings to their focus group results effectively. I do wish the authors would have included specific examples, derived from the focus group discussion and/or the literature, of certain things though, such as: (1) on page 7, left-hand side, in the paragraph about individualism/collectivism: examples of abstract versus concrete self-descriptions would be helpful, and (2) on page 8, left hand side of the page, an example of a vicarious memory statement would be helpful. 

 Another aspect of the work I found confusing and one that possibly takes away from the point of the article is the “P2” statement on page 8 (“I was drunk, I was inebriated.”). This statement makes sense, but it is still slightly confusing. Are the authors suggesting something in relation to gender is tied to a person who uses being intoxicated as a reason for why they didn’t act against the perpetrator? I assume this is the case based on the statement provided from “P1” right above it, but I think a better example would be more fitting – an example that more clearly demonstrates a gender role influence (i.e., a man, woman, any gender could say they were drunk as a reason to explain any subsequent information…it’s not clear to me how gender roles are associated with the statement made by “P2”).

 Finally, on page 7, on the ride side of the page, in the paragraph mentioning *focus* – more is needed to convince me that women will focus their testimony more on the person compared to the object (i.e., a weapon in this example) in relation to men. An example of sentences from women vs. men would be helpful, and a citation to support this argument is necessary because if this is true, the effect is surely in the literature. I also have an issue with the fact that the authors did not even acknowledge the weapon focus effect (Loftus, Loftus, & Messo, 1987) literature in this paragraph, considering one of their goals was to determine what should be considered when analyzing cultural differences in eyewitness memory. To my knowledge, there has not been substantial evidence of individual differences for the weapon focus effect. If there are individual differences related to any of the factors within the umbrella term, “culture” driving the presence of a weapon focus effect, the authors should mention it here. Furthermore, if women do focus their testimony more so on the person rather than the object, it should be noted this may not indicate anything about the accuracy of their memory compared to men. I argue this because the weapon focus effect is often found for peripheral details about the crime but doesn’t seem to appear as often in terms of details associated with the perpetrator and/or eyewitness identification performance.

 In terms of the study’s method, I have no issues with most of it except for a few things, such as the mention of the two focus group participants who provided written feedback about the manuscript because they couldn’t attend the focus group in person (page 5 under the participants section). Again, I have never conducted a focus study before, but providing written feedback seems qualitatively different than engaging in the focus group in real-time. I would like more detail regarding the type of feedback provided from the individuals who could not attend/how these individuals are considered participants rather than authors of the manuscript – were they given the transcript/audio of the conversation held between the focus group participants? Were they asked to simply add thoughts to the final manuscript? It’s unclear as to what their role was.

 Understandably, the focus group transcript is unavailable due to protecting the participants’ anonymity. I have no issue with this, but I would like to see more detail in terms of the methods and analysis, such as: (1) some kind of breakdown in terms of frequency of the subfactors present in the eyewitness interviews evaluated, (2) if some subfactors were not derived from the experts' comments about the interviews, and were instead only based on findings discovered during the critical literature review, I would like to see this pointed out, and (3) what specific cultures were represented in the eyewitness interviews? I don't think the manuscript ever mentions this information, and I think it is important to determine for two reasons - one being it would be necessary for replication and overlap versus no overlap in cultural backgrounds between the experts and the eyewitnesses may need to be addressed. Furthermore, the authors state that the eyewitness interviews used were those from males and females. I am okay with this, but later in the results section, they refer to gender roles but never biological sex (from what I can find), so clarification regarding how gender role information/data was discussed in the context of the eyewitness interviewee’s sex would be helpful if it occurred. Meaning, was this section based more on the critical literature review analysis or the analysis of the expert’s comments in the focus group discussion? As I mentioned above, I would like to see the section about gender roles on the right side of page 8 to be more cohesive in nature, meaning I don’t feel like the experts’ comments and the literature are blended well or creatively synthesized in this section like it is in pretty much all other sections devoted to other subfactors.

 In sum, this topic and this study will make a solid contribution to the literature, especially in the context of gathering useful information from the witness for purposes such as locating a suspect and then creating a lineup based on the description of the perpetrator provided by the witness. But the study also contains numerous foundations (a theoretical framework) from which many novel research studies can be developed. The Discussion section is strong, as it includes serious implications that the criminal justice system and eyewitness memory researchers should consider and provides clear examples/ideas on how to train interviewers with intercultural communication in mind. I would be willing to review this paper again after revision because I do believe the conclusions are adequate based on the results and highlights a huge gap in our approach to studying eyewitness/police procedures.

If applicable, is the statistical analysis and its interpretation appropriate?

Not applicable

Is the study design appropriate and is the work technically sound?

Yes

Is the work clearly and accurately presented and does it engage with the current literature?

Yes

Are the conclusions drawn adequately supported by the results?

Yes

Are sufficient details of methods and analysis provided to allow replication by others?

Partly

Are all the source data and materials underlying the results available?

Partly

Reviewer Expertise:

eyewitness memory and identification; face processing and recognition; experimental psychology - it was difficult for me to review and critique a focus study, as I have never completed one myself. I tried my best to only point out things that confused me with my little experience with qualitative research in mind.

I confirm that I have read this submission and believe that I have an appropriate level of expertise to confirm that it is of an acceptable scientific standard.

## References

[1] : Some facts about "weapon focus.". *Law and Human Behavior* .1987;11(1) : 10.1007/BF01044839 55-62 10.1007/BF01044839

[2] : What about N? A methodological study of sample-size reporting in focus group studies. *BMC Med Res Methodol* .2011;11: 10.1186/1471-2288-11-26 26 10.1186/1471-2288-11-26 21396104 PMC3061958
